# Diverging Drought Resistance of Scots Pine Provenances Revealed by Infrared Thermography

**DOI:** 10.3389/fpls.2016.01247

**Published:** 2016-08-31

**Authors:** Hannes Seidel, Christian Schunk, Michael Matiu, Annette Menzel

**Affiliations:** ^1^Department of Ecology and Ecosystem Management, TUM School of Life Sciences Weihenstephan, Technische Universität MünchenFreising, Germany; ^2^Institute for Advanced Study, Technische Universität MünchenGarching, Germany

**Keywords:** thermal imaging, water supply, aboveground dimensions, thermal indices, tissue temperature, CWSI, climate change

## Abstract

With recent climate changes, Scots pine (*Pinus sylvestris* L.) forests have been affected by die-off events. Assisted migration of adapted provenances mitigates drought impacts and promotes forest regeneration. Although suitable provenances are difficult to identify by traditional ecophysiological techniques, which are time consuming and invasive, plant water status can be easily assessed by infrared thermography. Thus, we examined the stress responses of 2-year-old potted Scots pine seedlings from six provenances (Bulgaria, France, Germany, Italy, Poland, and Spain) based on two thermal indices (crop water stress index and stomatal conductance index). Both indices were derived from infrared images during a 6-week drought/control treatment in a greenhouse in the summer of 2013. The pines were monitored during the stress and subsequent recovery period. After controlling for fluctuating environmental conditions, soil moisture or treatment-specific water supply was the most important driver of drought stress. The stress magnitude and response to soil water deficit depended on provenance. Under moderate drought conditions, pines from western and eastern Mediterranean provenances (Bulgaria, France, and Spain) expressed lower stress levels than those from both continental provenances (Germany and Poland). Moreover, pines from continental provenances were less resilient (showed less recovery after the stress period) than Mediterranean pines. Under extreme drought, all provenances were equally stressed with almost no significant differences in their thermal indices. Provenance-specific differences in drought resistance, which are associated with factors such as summer precipitation at the origin of Scots pine seedlings, may offer promising tracks of adaptation to future drought risks.

## Introduction

Scots pine (*Pinus sylvestris* L.) forests are sensitive to drought-related dieback. In a review of global forest mortality, Scots pine forests accounted for 40% (10 out of 25 cases) of all European die-off events (Allen et al., 2010). This situation might worsen in the future as climate change simulations propose increasing temperatures and decreasing local summer precipitation even in moderate scenarios (Kirtman et al., 2013). Seedlings and young trees are more vulnerable to stress (e.g., drought) than large trees, especially when not sheltered by dense canopies (Niinemets, 2010; Bussotti et al., 2015). This vulnerability might seriously impede forest regeneration. Thus, for successful forest management and climate change adaptation, the assisted migration of adapted tree species or the selection of suitable provenances might be necessary (Millar et al., 2007). This could be achieved by transferring seeds or plant material from drier and/or warmer climates to regions with similar future projected climates (Bussotti et al., 2015). Assisted migration might be especially appropriate for species with wide-ranging distributions and contrasting environments, such as *P. sylvestris* (Boratynski, 1991).

Scots pine provenances differ in their response to water availability. In a study conducted in Valais, Switzerland (Richter et al., 2012), the number of Mediterranean seedlings after a summer drought was twice the number of continental seedlings. Seedlings also differ in their shoot and/or height increments during drought periods (Taeger et al., 2013a, 2015). A dendroecological study revealed varying drought resistance among Scots pine provenances (Taeger et al., 2013b). Stomata-controlled leaf traits of *Pinus pinaster* (Fernández et al., 2000) and *P. halepensis* (Tognetti et al., 1997; Klein et al., 2013), such as stomatal conductance, transpiration rates, and intrinsic water-use efficiency, respond differently to water shortage in different provenances. However, no study has evaluated the provenance–drought interaction effects on the ecophysiological leaf traits in *P. sylvestris*.

Monitoring plant responses to climatic changes by ecophysiological techniques (e.g., water potential, xylem vulnerability to cavitation, stomatal conductance, transpiration rates, water use efficiency) is frequently time consuming and/or destructive. The applicability of these ecophysiological measurements in the field is generally reduced by limited accessibility to adult tree canopies. In contrast, crop breeding programs adopt non-invasive and high throughput techniques such as RGB imaging, chlorophyll fluorescence, thermal imaging, and imaging spectroscopy (Fiorani and Schurr, 2013). However, these techniques are mostly applied to morphologically simply structured organisms, e.g., *Arabidopsis*, or cereals and other crop plants.

When evaluating drought stress in plants, thermal imaging relates the actual surface temperatures of the leaves to their water availability. Plants interact with their aboveground environment by exchanging water, carbon, and energy, mostly through their stomata. One function of stomatal control is to maximize the photosynthetic gain while minimizing water loss through the leaves (Chaves et al., 2003; Jones, 2013). Meanwhile, the leaf tissue temperature depends on the stomatal conductance. As stomata close, the decreased transpiration reduces evaporative cooling and thus increases the leaf temperature (Raschke, 1960). Therefore, leaf temperature can be an indicator of stomatal closure and hence of water availability.

In recent years, the explanatory power of thermal images for drought stress responses has been improved by various approaches. Several thermal indices have been developed to normalize leaf surface temperatures under temporally changing environmental conditions. These indices have been linked to stem or leaf water potential and stomatal conductance (as reviewed in Maes and Steppe, 2012). Under field/outdoor conditions, thermal indices are especially recommended for constant (semi-) arid weather conditions as they have low variability under high vapor pressure deficits, so the changing weather conditions are relatively unimportant. Under temperate/moist conditions, the thermal indices are more problematic. They are also influenced by vegetation, canopy, and leaf characteristics. On the other hand, it is advantageous that thermal imaging can cover large spatial scales and efficiently catch the plant-to-plant variability in a single measurement (Maes and Steppe, 2012).

Most studies examining tree water status by thermal imaging have been conducted in orchards of almond, apple, citrus, olive or peach (Andrews et al., 1992; Sepulcre-Cantó et al., 2006; Ben-Gal et al., 2009; Wang and Gartung, 2010; García-Tejero et al., 2011; Gonzalez-Dugo et al., 2012; Zarco-Tejada et al., 2012; Agam et al., 2014; Virlet et al., 2014). In these studies, the thermal imaging discriminated between water-stressed and non-water-stressed individuals. The drought sensitivity of deciduous tree species has also been ranked by thermal imaging of their canopies (Scherrer et al., 2011). In general, these studies are hampered by the heterogeneity of orchard and forest trees and of the study sites themselves (Maes and Steppe, 2012). Apart from Leuzinger et al. (2010), thermal imaging of conifers is almost unreported in the literature; thus, comparison studies of conifers in different provenances by thermal indices are largely lacking.

We used thermal imaging to examine the drought stress responses of potted Scots pine seedlings from six provenances in a greenhouse experiment, assuming soil moisture as the most important driver. We investigated (i) whether Scots pines from different provenances differ in their stress responses, (ii) whether they respond differently to soil water deficit, and (iii) whether and to what extent the differences in thermal indices are explained by the plant dimension covariates. For a given water supply, the soil moisture in the pots might also depend on plant biomass (which differs among provenances), as larger individuals will probably have higher water consumption rates. Therefore, we additionally tested (iv) whether the stress levels under specific irrigation treatments differ among provenances.

## Materials and methods

### Experimental setup

#### Plant material

Scots pine seedlings were grown from seeds in a nursery in 2011 and were potted in April 2012. Pots had a volume of 3.l, were filled with peat substrate and placed in a greenhouse to conduct an extensive seasonal drought and warming experiment involving 10 provenances from all over Europe that started in 2013. Among this larger experimental setup, we randomly selected 48 2-year-old seedlings from six provenances. The climate conditions at the origin of the seeds are quite different (6–11°C annual mean temperature, 600–900 mm annual sum of precipitation), comprising Mediterranean-continental (Bulgaria), Mediterranean (Spain, France, Italy), and temperate-continental (Germany, Poland) sites (Table 1; see also Taeger et al., 2013a and Supplementary Figure 1). The heights and diameters of the seedlings before the experiment were similar across most provenances and treatments (Table 1; Supplementary Figure 2). However, the tree heights differed among the provenances (Kruskal-Wallis test, *p* < 0.001), being larger in the German and Polish provenances than in the Spanish (Dunn's test, *p* < 0.01), and the French one (Dunn's test, *p* < 0.05). There were no significant height differences across provenances between the two treatment groups, except in the control treatment where the German specimens were taller than the Spanish ones (Kruskal-Wallis test, *p* < 0.01). No significant height differences were observed among the treatments for all six provenances. Seedling stem diameters were also not significantly different between treatments and provenances except for the control treatment, in which the Italian specimens had larger diameters than the French specimens (Dunn's test following a Kruskal-Wallis test, *p* < 0.05).

**Table 1:** **Environmental conditions at the origin of Scots pine seeds; annual mean temperature (T), annual sum of precipitation (P) [1950–2000; obtained from the WorldClim data base (Hijmans et al., 2005)], aridity (P/PET) during the summer months (June to August; AI_summer_), replicates per treatment (N) and the means ± standard deviations of heights and diameters of 2-year old seedlings, grouped by provenance and treatment**.

**Provenance abbr**.	**Origin**	**Country**	**Lat**.	**Long**.	**Alt. (m)**	**T (°C)**	**P (mm)**	**AIsummer**	**Treatment**	**N**	**Height [mm]** ± **SD**		**Diameter [mm]** ± **SD**	
F 3	Prealpes du Sud	France	43°45′N	06°40′E	1185	7.8	675	0.65	Control	4	316.8 ± 71.4^ab^	A	6.8 ± 1.3^a^	A
									Drought	4	338.1 ± 40.5^a^		7.8 ± 0.7^a^	
PL 9	Suprasl	Poland	53°15′N	23°23′E	181	6.6	580	0.63	Control	3	430.1 ± 147.2^ab^	B	8.2 ± 1.1^ab^	A
									Drought	5	468.8 ± 95.3^a^		9.0 ± 1.1^a^	
ES 1	Alto Ebro	Spain	42°59′N	03°17′W	860	10.1	937	0.58	Control	5	285.2 ± 32.3^a^	A	8.3 ± 0.4^ab^	A
									Drought	3	272.3 ± 101.5^a^		7.2 ± 1.1^a^	
D 8	Mittel-/Ostdeutsches	Germany	53°04′N	13°29′E	75	8.4	571	0.55	Control	5	443.7 ± 35.4^b^	B	8.04 ± 0.5^ab^	A
	Tiefland								Drought	3	459.8 ± 104.6^a^		7.7 ± 1.0^a^	
BG 10	Garmen	Bulgaria	41°43′N	23°54′E	1300	6.2	657	0.54	Control	5	362.3 ± 63.3^ab^	AB	8.5 ± 1.7^ab^	A
									Drought	3	369.9 ± 68.6^a^		10.1 ± 2.2^a^	
I 4	Emilia Romagna	Italy	44°30′N	10°27′E	460	10.8	886	0.49	Control	5	366.3 ± 42.1^ab^	AB	9.2 ± 0.9^b^	A
									Drought	3	360.1 ± 23.7^a^		8.2 ± 1.1^a^	

Provenances are arranged by decreasing AI_summer_. Different letters subscripting the height and diameter values indicate significant differences: Capital and lower-case letters refer to overall differences between provenances and the within-treatment (control or drought) differences between provenances, respectively. No significant differences were found between the treatments in each provenance.

#### Drought treatment

**Figure 1:**
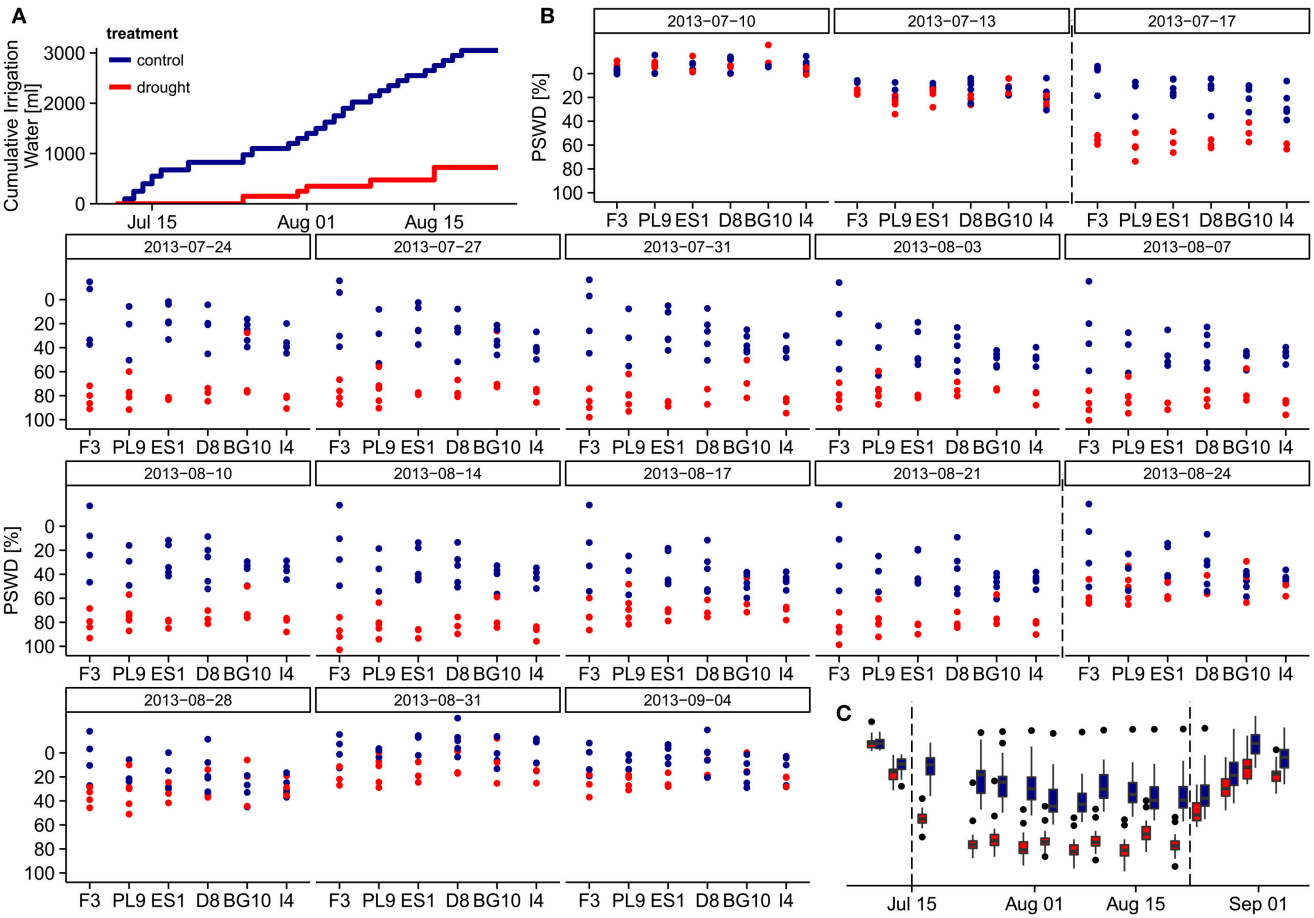
**Water relations during the experiment**. **(A)** Cumulative irrigation water per individual under different treatments during the drought treatment period (July 13th to August 21st). **(B)** Percent soil water deficit (PSWD) of the individuals from different provenances during the study period. On the first measuring day (July 5th), the pots were fully saturated and the PSWD was zero, so this day is excluded. **(C)** PSWD in the control and drought treatments throughout the study period. Dashed lines indicate start and end of the stress period. PSWDs > 0% indicate over-saturated soil. Provenances are abbreviated as follows: F3 France, PL9 Poland, ES1 Spain, D8 Germany, BG10 Bulgaria, and I4 Italy.

Automated dripping irrigation allowed four water treatment groups in the larger experimental setup, but we implemented just two treatments under the time constraints of this study. Twenty-seven of the selected individuals assigned to the control group, and 21 were subjected to a summer drought from July 11th to August 21st 2013. In the drought treatment, irrigation was initially intermitted and only small amounts of water were added afterwards forcing the soil moisture to oscillate around the permanent wilting point. During this 42-day period each individual in the control group received 3050 ml (i.e., 190 mm) water, while individuals in the drought treatment received only 725 ml (i.e., 45 mm, Figure 1A). On August 22nd, all pots were saturated with water; in the subsequent recovery period until September 4th, all specimens were again well-watered with identical amounts of water. We weighted each of the 48 pots on each measuring day, and calculated the percent soil water deficit (PSWD) as the difference between the pot weight at field capacity on July 5th and the actual weight of the pots divided by the absolute water content at field capacity (see Figures 1B,C). The absolute water content of the pots at field capacity was derived from water retention curves following the pressure plate method by Richards (1941) and was estimated as 40% at 10 kPa soil-water matrix potential.

#### Moisture levels during experiment

On July 5th, all pots were fully saturated and their calculated percent soil moisture deficits (PSWDs) were zero (therefore, the first measuring day is omitted from Figures 1B,C). During the drought treatment period (from July 11th to August 21st), individuals in the drought group received only ~24% of the irrigation water added to the control group (Figure 1A). As revealed in the overarching larger experiment, this drought treatment corresponded to conditions around the permanent wilting point. Seven days from the start of treatment (July17th), PSWD differences between the treatments became significant (Wilcoxon test, *p* < 0.001; Figure 1B). We thus define the period from July 17th until August 21st as the stress period. We could not detect any significant differences in PSWD across provenances within each treatment (see Supplementary Figure 2C for mean PSWDs during the stress period), although the individuals of some provenances differed in height and diameter, likely causing unequal water depletion in the pots. In both treatment groups, the PSWD decreased at the beginning of the stress period and stabilized after approximately 1 week; however, PSWD of the drought treatment group remained significantly lower until August 21st (Figure 1C). These differences partly remained during the recovery period (from August 22nd to September 4th; measuring days 14–17), although the pots were fully saturated with water on August 22nd. Because the PSWDs of the two treatment groups were not always fully separated, we modeled the stress response in two ways; the first based on the absolute PSWD, the second based on drought treatment vs. control treatment referring to different water supply scenarios (see subsection Statistical analysis).

### Thermal indices

The plant surface temperatures can be related to drought stress and plant water status by several methods (reviewed in Maes and Steppe, 2012). To account for the changing environmental conditions, the surface temperatures must be normalized by reference temperatures (see Reference surfaces and plant monitoring platform). We used two thermal indices; the crop water stress index (CWSI) and stomatal conductance index (Ig).

Jones (1999) proposed the CWSI as a modification of Idso et al.'s (1981) formulation. The CWSI is known to mirror stomatal conductance and the leaf and stem water potentials. It normalizes the leaf surface temperatures by the surface temperatures of wet (*T*_*wet*_) and dry (*T*_*dry*_) references, where *T*_*wet*_ represents a fully transpiring leaf and *T*_*dry*_ a non-transpiring leaf. The CWSI is calculated as

(1) $$\begin{array}{l} CWSI=\frac{T_{canopy}\;-\;T_{wet}}{T_{dry}\;-\;T_{wet}} \end{array}$$

*I*_*g*_ employs the same variables as *CWSI* but is linearly related to the stomatal conductance (Jones, 1999). Thus, *Ig* is a linear function of the stomatal opening:

(2) $$\begin{array}{l} I_{g}=\frac{T_{dry}\;-\;T_{canopy}}{T_{canopy}\;-\;T_{wet}} \end{array}$$

Higher *CWSI* and lower *Ig* values indicate higher surface/tissue temperatures and thus stomatal closing.

### Reference surfaces and plant monitoring platform

**Figure 2:**
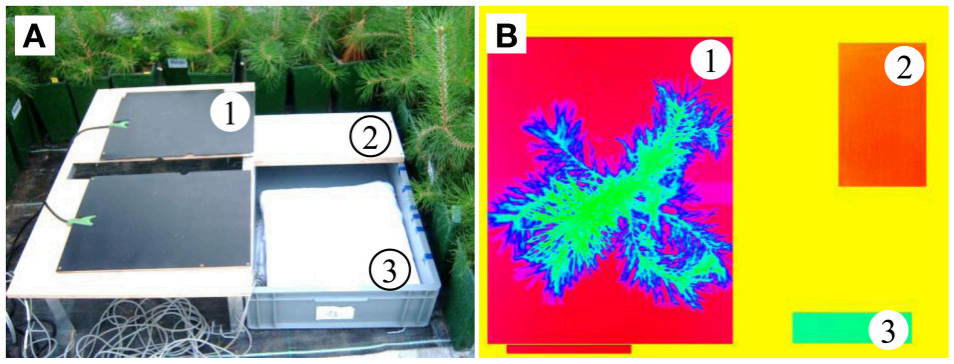
**(A)** Plant monitoring platform and **(B)** a thermal image, showing (1) the heating plates, (2) dry reference, (3) wet reference, and a pine located between the heating plates.

**Figure 3:**
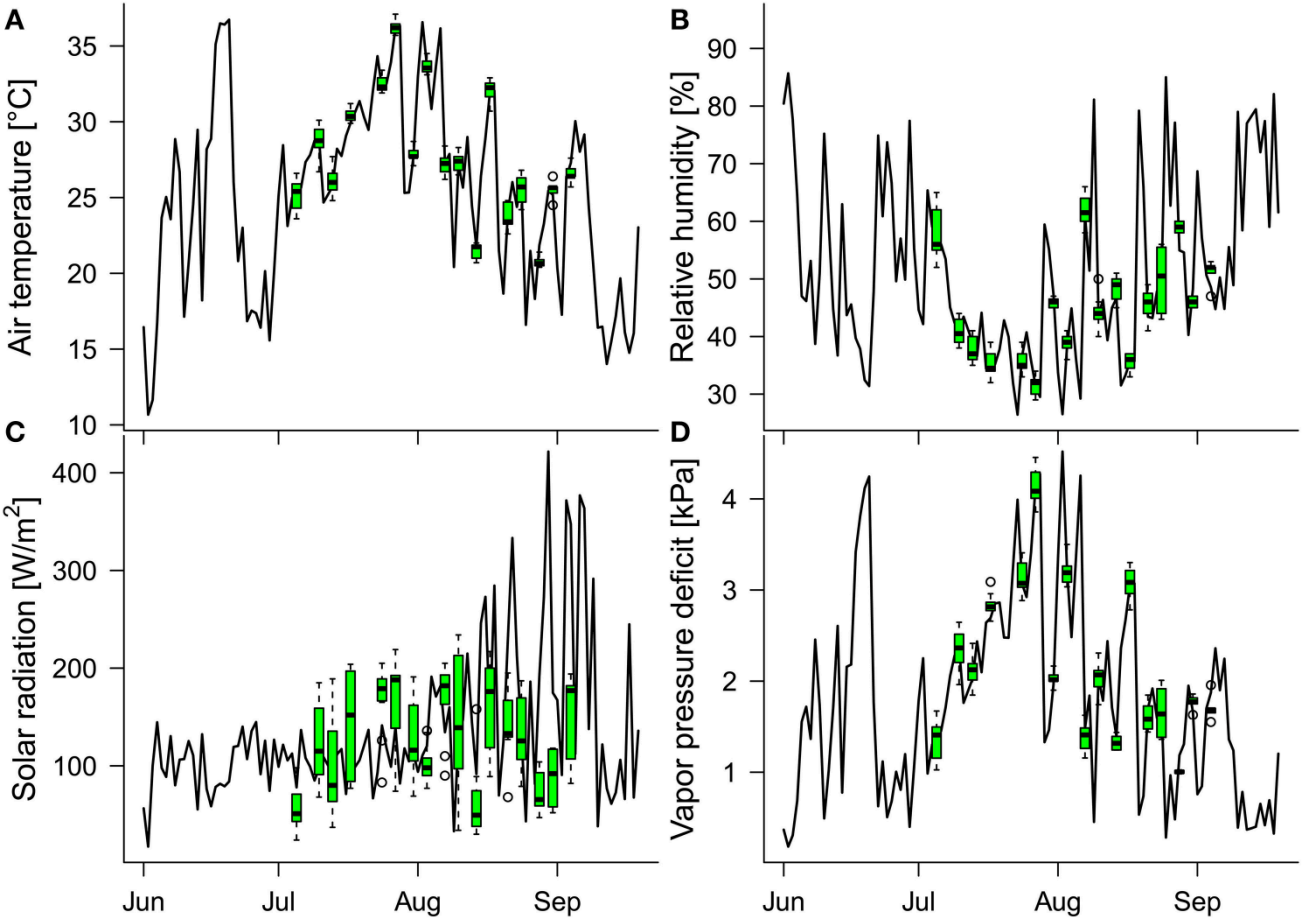
**Daily means of the in-greenhouse meteorological conditions measured between 11 a.m. and 3 p.m. (time window of thermal image acquisition on the measuring days): (A) air temperature, (B) relative humidity, (C) solar radiation, and (D) vapor pressure deficit**. Green boxplots show the distributions of meteorological data (11 a.m. to 3 p.m.) of the respective variables on the measuring days. Because the greenhouse is protected from overheating by automated shading, the mean solar radiation is low during midsummer and increases toward late summer (when the outside temperatures and solar radiation decrease).

As suggested in Meron et al. (2003) and Möller et al. (2006), we calculated the abovementioned thermal indices using artificial reference surfaces (ARSs), which are included in each picture (see Meteorological data and Figure 2). To mimic the maximum transpiring surface, we wrapped a white cotton fabric around a styrofoam board floating in a water-filled plastic box (wet reference). A non-transpiring leaf was represented by an opal white laminated fiberboard (dry reference) mounted on the plastic box (Figure 2). Jones (1999) used wet and dry leaves as the reference surfaces, but here we chose the ARSs because the thin needles of conifers (unlike leaves) easily dry out under the high greenhouse temperatures (Figure 3A). Additionally, the temperature information in the pixels of the reference needles might become mixed with that of non-reference needles in the background. Unfortunately, this approach might be sensitive to changing environmental conditions because the short-wave absorptances and heat capacities differ between needles and reference targets. For these reasons, environmental variables were incorporated as control covariates in the statistical models (see Statistical analysis). In addition to the wet and dry ARSs, two black painted electric heating plates with a mean temperature of 40°C were horizontally attached to a support frame. These created a strong contrast to the plant tissue and totally masked the pot and soil in the images. A small gap in the middle edge of the plates prevented squeezing of the trunk. The handling time of taking an individual tree to the monitoring platform, mounting it into the platform and capturing up to three thermal images was approximately 3 min.

### Thermal imaging

Thermal images were acquired by a thermal infrared camera (VarioCam hr inspect 780, Infratec, Dresden Germany) with a resolution of 1280 × 960 infrared pixels using the optomechanical resolution enhancement. The thermal resolution within the images was below 0.08 K at 30°C and the absolute measurement accuracy was ± 1.5 K. The emission coefficient was set to 1 during the image acquisition and was later corrected using the emission coefficients calculated by the quotients of the contact thermocouple temperatures and the thermal image-derived temperatures of the reference surfaces and pine seedlings (performed in a dark chamber under temperature and relative humidity control and in the greenhouse, respectively). The resulting emission coefficients of the wet reference, dry reference and pines were 0.95, 0.93, and 0.90, respectively. The calculated emissivity can be considered as the apparent emissivity since we did not measure background temperature. The errors caused by this omission should be negligible inside the greenhouse, which was constantly shaded for the thermal imaging, largely corresponding to cloudy conditions (see Meteorological data, Maes and Steppe, 2012). Thermal images were taken approximately twice a week from July 5th to September 4th, leading to 17 measuring days. Ten of these days constituted the stress period with clear PSWD effects, and 4 days followed the summer drought treatment (see Figure 1B for exact dates). During the image acquisition (between 11 a.m. and 3 p.m.), the greenhouse was shaded to reduce the possible influence of variable solar radiation (e.g., due to scattered clouds). The camera was vertically mounted at 2.5 m above the monitoring platform attached to the scaffolding of the greenhouse. On every measuring day, each of the 48 pines was photographed 2 or 3 times (rarely once due to technical problems), together with the reference surfaces.

In the subsequent image analysis, the plant tissue was separated from the (heated) background, and interferences at the needle edges (where single pixels were mixtures of plant and background temperatures) were additionally removed. To automate the image processing, a script was written in Fiji (Schindelin et al., 2012). Images were processed by the following steps. First, the image was sharpened using the command “Unsharp Mask” with a radius of 2 and a mask weight of 0.9. From this sharpened image, two masks were created, one to remove the background (using auto thresholding based on the intermodes algorithm), the other to remove the edges (using the “Find Edges” command). The median temperature of the plants' canopy and the dry and wet references in each image were calculated from the remaining pixels. The respective mean daily temperatures were then determined from the multiple (1–3) images acquired on each measuring day. Finally, the thermal indices were calculated from the mean temperatures per measuring day and used in subsequent statistical analysis.

### Meteorological data

During the thermal image acquisition, an air temperature (T) and relative humidity (RH) sensor (HOBO U23 Pro v2, Hobo®, Onset Computer Corporation, Bourne, MA) was placed next to the plant monitoring platform. Data were recorded in 1-min intervals. In the greenhouse, the air temperature, relative humidity and solar radiation were measured at 10-min intervals throughout the whole study period by a meteorological weather station (Davis Vantage Pro2 Plus™, Davis Instruments, Hayward, CA). We matched the meteorological and thermal image data with their nearest temporal counterparts. The vapor pressure deficit (VPD), defined as the difference between saturation vapor pressure (e_s_) and actual vapor pressure (e_a_), was calculated after Allen et al. (1998) with T and RH as the input variables.

The daily mean values of the air temperature, relative humidity, solar radiation, and vapor pressure deficit, collected at 10-min intervals between 11 a.m. and 3 p.m. (the time window of the thermal image acquisition on measuring days), varied during the study period (July 5th to September 4th), within the ranges 16.6–36.6°C, 26.4–85.0%, 33.0–421.7 W/m^2^, and 0.3–4.5 kPa, respectively (Figure 3). The minimum and maximum air temperature, relative humidity, solar radiation and vapor pressure deficit during the image-acquisition time over the 17 measuring days were 20.4 and 37.1°C, 29 and 66%, 24 W/m^2^ and 234 W/m^2^, and 1.0 kPa and 4.5 kPa, respectively. Thus, the conditions during measuring times well represented the indoor conditions at noon over the course of the study period except for the solar radiation, which was mostly determined by the additional shading in the greenhouse (generally throughout June and July, and on the measuring days after mid-August).

### Statistical analysis

We analyzed the effect of PSWD on thermal indices over the whole study period (from July 5th to September 4th). The analysis was performed by linear mixed-effects models (nlme; Pinheiro et al., 2016) implemented in R version 3.2.2 (R Core Team, 2015). Full models were constructed by adding the covariates provenance as factorial dummy variable, heights, and diameters of seedlings at the beginning of the experimental period, air temperature, relative humidity, vapor pressure deficit, and solar radiation and the two-way interactions of PSWD with provenance, air temperature, relative humidity, and vapor pressure deficit. Because the PSWD varies nonlinearly with the thermal indices, it was added as a linear and quadratic term to the models. All covariates besides PSWD were centered on their means (by subtracting their respective means from the discrete variable values) for easier interpretation of interaction effects. As time and PSWD were strongly correlated, they cannot be included simultaneously due to collinearity; hence, no time variable was included in the PSWD-based models. To account for the repeated measurements of individuals during the experiment, we included the individual trees as random variables.

In a first step of model selection we either chose air temperature plus relative humidity or vapor pressure deficit as covariates based on the AIC (Akaike Information Criterion) of the respective models, since these variables were strongly correlated. The full model is mathematically expressed as

(3) $$\begin{array}{l} Index_{i,j}=\beta_{0}\;+\;b_{0,i}\;+\;\gamma_{1}Provenance_{i}\;+\;\beta_{1}PSWD_{i,j}\\ \;+\;\beta_{2}PSWD_{i,j}^{2}\;+\;\beta_{3}Height_{i}\;+\;\beta_{4}Diameter_{i}\\ \;+\;\beta_{5}VPD_{i,j}\;+\;\beta_{6}Radiation_{i,j}\;+\;\gamma_{2}Provenance_{i}\\ \;*\;PSWD_{i,j}\;+\;\gamma_{3}Provenance_{i}\;*\;PSWD_{i,j}^{2}\;+\;\beta_{7}VPD_{i,j}\\ \;*\;PSWD_{i,j}\;+\;\epsilon_{i,j} \end{array}$$

with random intercepts $b_{0,i}\sim N\left(0,\sigma_{0}^{2}\right)$ and errors $\epsilon_{i,j}\sim N\left(0,\sigma_{\epsilon }^{2}\right)$. *Index*_*i, j*_ is the thermal index of tree *i* (= 1 …48) at measurement *j* (= 1 …17). The meteorological variables (in this case, the VPD and radiation) also vary from tree to tree, because they were measured sequentially on the measurement day, and altered throughout the course of the day. As the six provenances were modeled using dummy variables, each of γ_1_, γ_2_, andγ_3_ is a five-dimensional coefficient vector of the dummy regressors.

To simplify the full models, we evaluated the importance of the explanatory variables/interactions using the drop1 function (stats; R Core Team, 2015). Any terms that did not improve the models' explanatory power were excluded (Table 2). The R^2^ of the final models was computed by the r.squaredGLMM function (MuMIn; Barton, 2015, Table 3). To avoid heteroscedacity and non-normal distribution of the residuals, we examined the diagnostic plots and applied variance function structure classes. The Ig was square-root-transformed to meet these criteria. Provenances were compared by a pairwise *post-hoc* test using the glht function (multcomp, Hothorn et al., 2008) comparing contrasts with the Tukey's range test. By this test, we also compared the provenances under additional PSWD scenarios (0%, 50%, and 100% PSWD) after centering the PSWD values on these thresholds and refitting the models. To check the different stress behaviors of provenances in relation to PSWD, we tested the coefficients of the linear and quadratic terms of the PSWD–provenance interaction with the glht function.

**Table 2:** **Variables included in the final linear mixed models evaluating the effect of percentage soil water deficit (PSWD) on the thermal indices CWSI and Ig, respective effect sizes (Estimate) and *p*-values extracted from the summary table of the models**.

	**CWSI (*R*^2^ = 0.64)**		**Ig (*R*^2^ = 0.62)**	
	**Estimate**	***P*-value**	**Estimate**	***P*-value**
Provenance	X		X	
PSWD	−2.49	< 0.001	4.39	< 0.001
PSWD^2^	1.61	< 0.001	−2.91	< 0.001
Provenance × PSWD	X		X	
Provenance × PSWD^2^	X		X	
VPD	0.044	< 0.001	−0.081	< 0.001
Radiation	0.00032	< 0.001	−0.00061	< 0.001

Effect sizes of categorical variables (provenance) and interaction terms involving this variable are not shown, but their contribution is indicated by X. The PSWD contribution involves a linear (PSWD) and a quadratic (PSWD^2^) component. In the model fittings, the vapor pressure deficit (VPD) and solar radiation were centered on their means. The Ig was square-root-transformed.

**Table 3:** ***P*-values of the pair-wise comparisons (Tukey's range test of contrasts) of the provenances' response to PSWD estimated by linear mixed-effects models**.

	**F3**	**PL9**	**ES1**	**D8**	**BG10**	**I4**
F3		0.66/0.49	**<0.05/<0.01**	0.89/0.61	0.16/0.17	0.59/0.59
PL9	0.53/0.28		**<0.05/<0.05**	0.64/0.89	0.37/0.49	0.89/0.89
ES1	**<0.01/<0.01**	**<0.05/0.14**		**<0.05/<0.01**	0.51/0.29	0.06/ **< 0.01**
D8	0.85/0.49	0.53/0.71	**<0.01**/0.05		0.16/0.44	0.58/0.95
BG10	0.22/0.23	0.53/0.71	0.27/0.28	0.28/0.59		0.49/0.44
I4	0.53/0.64	0.85/0.56	**<0.05/<0.05**	0.63/0.71	0.53/0.44	

P-values are listed for the coefficients of linear and quadratic components of PSWD (PSWD/PSWD^2^). The upper-right and lower-right triangles display the CWSI and Ig results, respectively. Provenances are abbreviated as follows: F3, France; PL9, Poland; ES1, Spain; D8, Germany; BG10, Bulgaria and I4 Italy. Bold values indicate significance at a level of 0.05.

Differences in thermal indices between the treatments (water supplies) and among the provenances, and in their corresponding ability of the pines to recover from the water stress, were separately analyzed over the stress period (measuring days 4–13, July 17th until August 21st) and the recovery period (measuring days 14–17, August 24th until September 4th). Here we fitted and simplified the linear mixed models as described above. The factorial dummy variable treatment (control/drought) was used rather than the PSWD, and the two-way interactions of treatment with height and diameter were added to the initial full model. As the PSWD changes over time in both treatments and study periods, we added a time variable (number of days since the observations started) as a control covariate with a linear and a quadratic term. Based on the AIC, we selected the VPD and air temperature/relative humidity as covariates. The full model is mathematically expressed as

(4) $$\begin{array}{l} Index_{i,j}=\beta_{0}\;+\;b_{0,i}\;+\;\gamma_{1}Provenance_{i}\;+\;\beta_{1}Treatment_{i}\;+\;\beta_{2}t_{j}\\ \;+\;\beta_{3}t_{j}^{2}\;+\;\beta_{4}Height_{i}\;+\;\beta_{5}Diameter_{i}\\ \;+\;\beta_{6}Temperature_{i,j}\;+\;\beta_{7}RelativeHumidity_{i,j}\\ \;+\;\beta_{8}Radiation_{i,j}\;+\;\gamma_{2}Provenance_{i}\;*\;Treatment_{i}\\ \;+\;\beta_{9}Treatment_{i}\;*\;t_{j}\;+\;\beta_{10}Treatment_{i}\;*\;t_{j}^{2}\\ \;+\;\beta_{11}Treatment_{i}\;*\;Height_{i}+\;\beta_{11}Treatment_{i}\\ \;*\;Diameter_{i}\;+\;\beta_{12}Treatment_{i}\;*\;Temperature_{i,j}\\ \;+\;\beta_{13}Treatment_{i}\;*\;RelativeHumidity_{i,j}\;+\;\epsilon_{i,j} \end{array}$$

where *Treatment*_*i*_ is 0 if tree *i* received the dry treatment, and 1 for the control group. *t*_*j*_ is the number of days after the start of the experiment (July 5th) at measurements *j* (= 4 …13) for the stress period and *j* (= 14 …17) for the recovery period. Employing the glht function, we again tested the differences among provenances and between treatments in pairwise *post-hoc* tests, comparing contrasts with the Tukey's range test.

Additionally, we analyzed the response magnitudes of the provenances between treatments during the stress period. The mean index values were calculated for each individual. The response magnitude was calculated as the pairwise difference in particular indices between each individual of the drought group and all other individuals of the control group. Differences in response magnitude, tree height and diameter were analyzed by the Kruskal–Wallis test (stats; R Core Team, 2015) and by Dunn's test for multiple comparisons (FSA; Ogle, 2015).

All *p*-values for multiple comparisons were corrected by the false discovery rate (FDR) method.

## Results

### Relationship between thermal indices, PSWD, and provenances

The two thermal indices were significantly related to PSWD, provenances and meteorological control parameters. Excluding some meteorological control covariates (temperature, relative humidity) and plant biomass covariates (diameter, height) from the full models, the final models resulted in better fits in terms of lower AIC-values and explained a proportion of variance of 0.64 and 0.62 for CWSI and Ig, respectively (Table 2).

**Figure 4:**
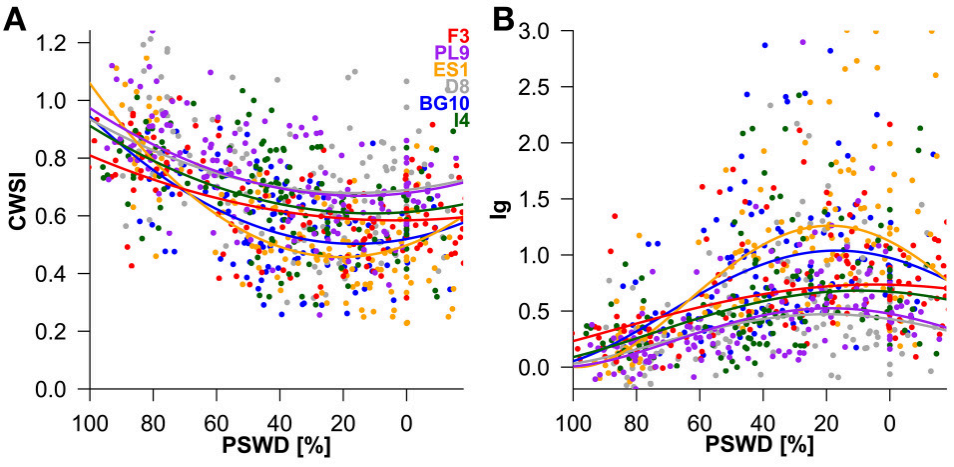
**Influence of percent soil moisture deficit (PSWD) on CWSI (crop water stress index; (A) and Ig (stomatal conductance index; (B) during the study period (July 5th to September 4th)**. Dots and lines represent the measured daily mean values of the individual Scots pine seedlings and the modeled estimates, respectively. PSWD responses of provenances are shown for mean VPD and radiation conditions. Provenances are abbreviated as follows: F3 France, PL9 Poland, ES1 Spain, D8 Germany, BG10 Bulgaria, and I4 Italy.

To interpret the signs of the model variables (Table 2), we must remember that CWSI and Ig increase and decrease at higher stress levels, respectively. In both thermal indices, higher stress levels were linked to higher PSWD, indicating lower soil-water availability (Table 2; Figure 4). However, provenance and the its interaction with PSWD were also included in the models of the thermal indices (Table 2). The Bulgarian and especially the Spanish provenance showed a pronounced stress minimum (PSWD = 15–20%; see Figure 4). Although responses of provenances to PSWD were varying, significant differences were detected only in the Spanish provenance. Pines in this provenance were much more sensitive to increasing water deficit than all provenances other than the Bulgarian one (Table 3; Figure 4). Increasing VPD and radiation also increased the stress levels (either by natural response of the stomata and leaf surfaces or because the ARS-derived indices were sensitive to the changing environmental conditions); Figure 4 displays the responses of the different provenances to PSWD, for the mean VPD and radiation conditions. Note that all dimensional variables (seedling height and diameter) were excluded from the final model because they added no further explanatory power aside from provenance.

### Discrimination of provenances under different soil moisture scenarios

Under well-watered conditions (PSWD = 0%) the provenances from Spain and Bulgaria were less stressed than those from Poland and Germany (*p* < 0.05; Supplementary Table 1). Marginal differences were observed between pines from France and Germany and between pines from Italy and Spain (*p* < 0.1; data not shown). At medium soil moisture (PSWD = 50%) the Bulgarian and Spanish seedlings were less stressed than the German and Polish seedlings and the Spanish provenance performed better than the Italian provenance (*p* < 0.05). Additionally, the CWSI was marginally different between the French and Spanish seedlings and between Italian and Bulgarian provenances (*p* < 0.1; data not shown). Under severe drought conditions (100% PSWD), only the two most extreme provenances (France and Spain) showed significant differences in both thermal indices (*p* < 0.01). The Ig significantly differed between pines from Poland and France (*p* < 0.05; Supplementary Table 1).

### Variation of stress level with provenance, treatment (water supply), and seedling dimensions

The immediate drought stress during the stress period and the conditions during the recovery period, were assessed for both treatments (defined by their water supply i.e., the amount of irrigation water) by the thermal indices. For both CWSI and Ig, the explanatory power of the corresponding linear mixed models was lower during the stress period (both indices = 0.63) than during the recovery period (0.74 and 0.75, respectively, Table 4).

**Table 4:** **Variables included in the final linear mixed models evaluating the influence of the drought treatment (defined by water supply) on the thermal indices CWSI and Ig during the stress period (measuring days 4–13, July 17th to August 21st) and the recovery period (measuring days 14–17, August 24th to September 4th)**.

	**Stress period**			
	**CWSI (*R*^2^ = 0.63)**		**Ig (*R*^2^ = 0.63)**	
	**Estimate**	***P*-value**	**Estimate**	***P*-value**
Treatment	−0.23	**<0.001**	0.43	**<0.001**
Provenance	X		X	
Treatment × Provenance	X		X	
Height	−0.000034	0.88	0.00014	0.78
Height × Treatment	0.00074	**<0.05**	−0.0013	**0.05**
Diameter	−0.03	**<0.05**	0.056	**<0.05**
Time	0.42	**<0.01**	−0.54	0.07
Time2	−0.42	**<0.01**	0.68	**<0.05**
T	0.0086	**<0.01**	−0.013	**<0.05**
T × Treatment	−0.012	**<0.01**	0.02	**<0.01**
RH	−0.0007	0.69	0.0002	0.94
RH × Treatment	−0.01	**<0.001**	0.02	**<0.001**
Radiation	0.00025	**<0.05**	/	/
	**Recovery period**			
	**CWSI (*R*^2^ = 0.74)**		**Ig (*R*^2^ = 0.75)**	
	**Estimate**	***P*****-value**	**Estimate**	***P*****-value**
Treatment	−0.11	**<0.001**	0.23	**<0.001**
Provenance	X		X	
Time	−0.01	**<0.001**	0.018	**<0.001**
T	0.024	**<0.001**	−0.052	**<0.001**
Radiation	0.00086	**<0.001**	−0.0015	**<0.001**

The effect sizes (Estimates) and p-values are extracted from the summary table of the models. The effect sizes of categorical variables with more than two levels (provenance) and interaction terms containing this variable, are not shown, but their contribution is indicated by X. The models were fitted with the continuous variables centered on their means. The Ig was square-root-transformed. T and RH denote air temperature and relative humidity, respectively. Bold values indicate significance at a level of 0.05.

During the stress and recovery periods, the respective final models of CWSI and Ig included provenance, time and treatment. As expected, stress was increased by the drought treatment and continued to increase throughout the stress period, then declined over the recovery period. In contrast, the sets of plant specific (dimension, provenance) and meteorological covariates explaining the variation in thermal indices differed between stress and recovery periods (Table 4).

**Figure 5:**
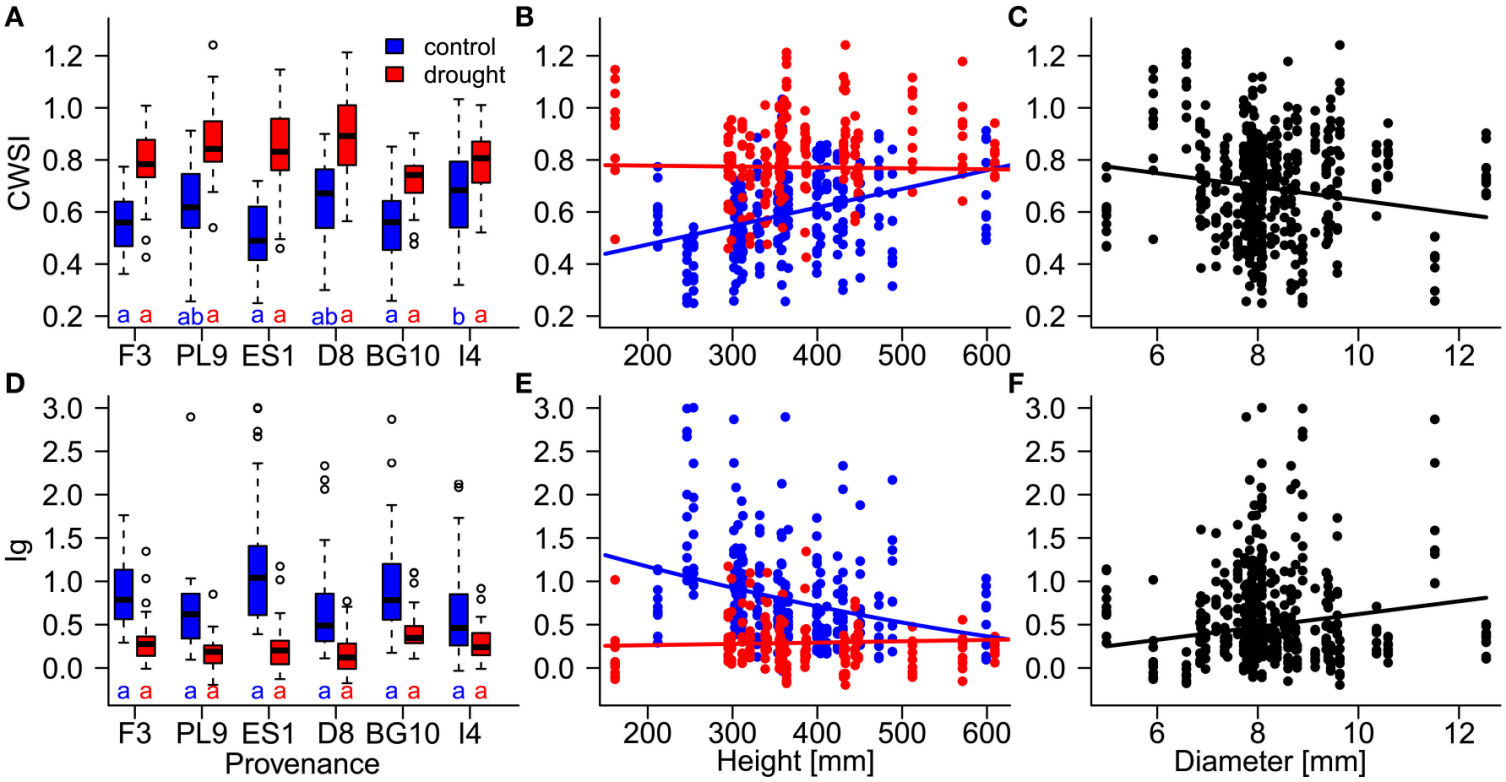
**Influences of (A,D) provenances, (B,E) height, and (C,F) diameter on the resilience of individual Scots pine trees to drought stress, evaluated by the thermal indices CWSI (crop water stress index; A–C) and Ig (stomatal conductance index; D–F) during the stress period (July 17th to August 21st)**. Influences of provenance and height differed between the control (blue) and drought treatment (red). Provenances with the same letters below their boxplots **(A,D)** show no significant differences between the control treatment (blue) and drought treatment (red; at the 5% significance level). Provenances are abbreviated as follows: France (F3), Poland (PL9), Spain (ES1), Germany (D8) Bulgaria (BG10), and Italy (I4).

Diameter was negatively associated with stress during the stress period, but was unimportant in the models of the recovery phase (Table 4; Figures 5B,C). The influence of seedlings' height on the thermal indices depended on the water supply treatment (Table 4; Figures 5B,C). Specifically, the stress levels increased with height in the control treatment but were independent of height in the drought treatment. Equally, height was unimportant in the recovery period models.

**Figure 6:**
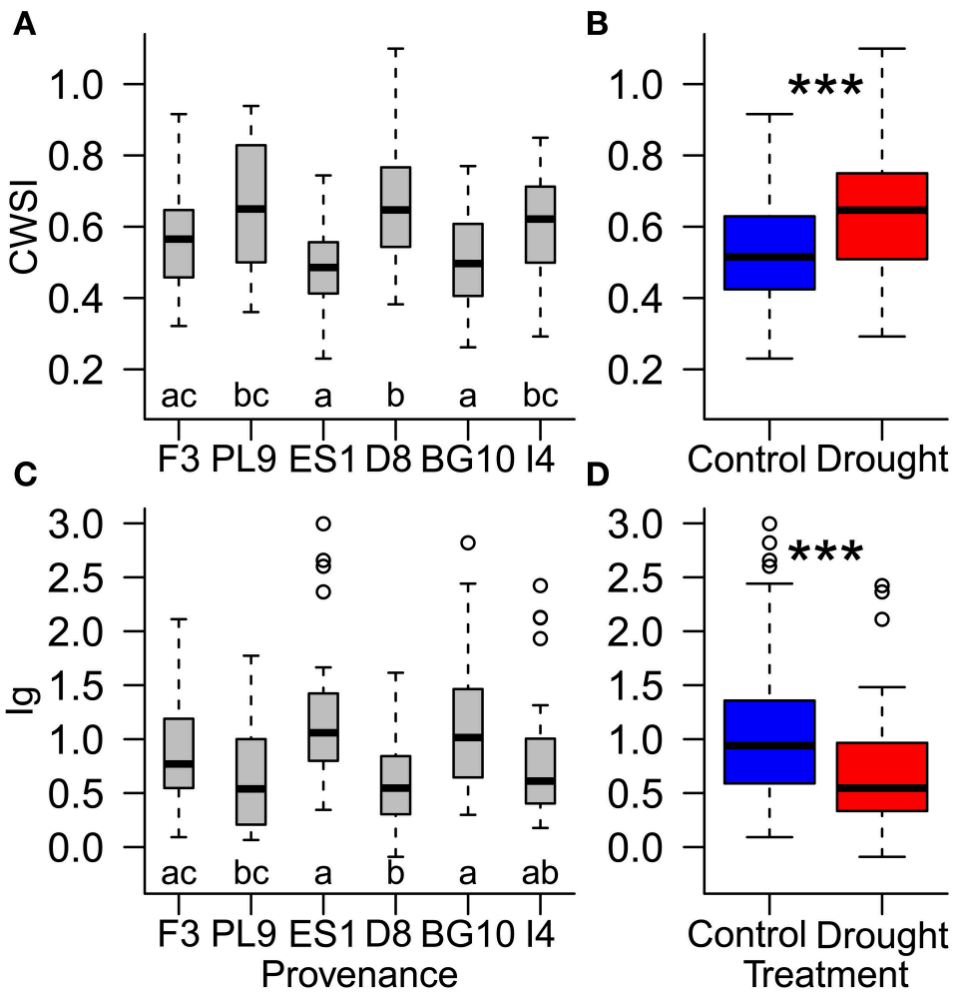
**Influence of (A,C) provenances and (B,D) water supply treatment on the resilience of individual Scots pine trees to drought stress, evaluated by the thermal indices CWSI (crop water stress index; A,B) and Ig (stomatal conductance index; (C,D) during the recovery period (August 24th to September 4th)**. Different letters **(A,C)** and asterisks **(B,D)** indicate significant differences. Provenances are abbreviated as follows: France (F3), Poland (PL9), Spain (ES1), Germany (D8), Bulgaria (BG10), and Italy (I4).

During the stress period, the effect of provenance on the thermal indices depended on the treatment (Table 4; Figure 5), whereas during the recovery period, the treatment and provenance influenced the thermal indices without interacting with each other (Table 4; Figure 6). In each provenance, the seedlings were significantly more stressed in the drought than in the control group during the stress period (*p* < 0.01, Supplementary Table 2). The exception was the Italian provenance, where high stress levels were already observed in the control group (Figure 5). Although the treatment differences under stress decreased by ~50% during the recovery period (Table 4), the overall differences between treatments persisted in the recovery period (Figures 6B,D; Supplementary Table 3). During the stress period, the provenance differences were not significant in the highly stressed drought treatment group. However, in the control group, whose PSWD levels imposed moderate stress during this period, the CWSI levels indicated more stress in the Italian provenance than in provenances from France, Spain and Bulgaria. However, the Ig did not capture these differences at a significance level of 0.05 (Figures 5A,D; Supplementary Table 2).

During the recovery period, provenances showed equal stress patterns in both treatment groups, because the provenance and treatment interaction was not significant in the statistical model (Table 4). According to both thermal indices, the provenances from Spain and Bulgaria were less stressed than those from Poland and Germany, and the French provenance was less stressed than the German provenance (Figures 6A,C; Supplementary Table 3). Furthermore, the CWSI showed higher stress levels in the Italian seedlings in than the Spanish and Bulgarian ones. Although the seedlings recovered, the significant difference between the control and experimental groups persisted into the recovery period, indicating some post-drought stress (Table 4; Figures 6B,D).

### Response to water supply treatments

The treatment effect (water supply) during the stress period clearly separated the individuals of almost every provenance. The exception was the Italian provenance, in which the negative group differences indicated overlap of the stress levels of both treatment groups (Supplementary Figure 3). The differences between treatments exhibited similar patterns for both thermal indices. The Spanish and Italian provenances demonstrated the strongest and weakest response to water supply, respectively. During the stress period, the response to the water supply treatment was stronger in the Spanish pines than in the Polish, Bulgarian, and Italian pines, as shown by both indices (Supplementary Table 4). The CWSI and Ig revealed further differences in the water supply response; specifically, the Italian seedlings demonstrated weaker CWSI response than the French, Polish, and German seedlings, and the Ig response to water supply was higher in Spanish than in German pines (Supplementary Table 4).

## Discussion

### Methodological considerations of thermal imaging

When performing thermal imaging under non-100% controlled conditions, several limitations must be considered (Prashar and Jones, 2014). Variations in environmental conditions affect the leaf temperature, so reference surfaces are required. Here we replaced wet and dry Scots pine needles with ARSs (see Materials and Methods) instead of using wet and dry Scots pine needles which would have had similar spectral and physical properties to the target leaves (Jones, 1999). Nevertheless, the ARS performance should be adequate for the following reasons: (1) the emissivity of each target surface was determined; (2) the greenhouse was shaded before and during image acquisition, meaning that all thermal images were acquired under low levels of incoming solar radiation; (3) on most measurement days, the T, RH, and VPD varied within a narrow range, suggesting that the errors introduced by differing heat capacities of the targets are also small; (4) when comparing indices across provenances and treatments, these errors can be partially compensated by incorporating meteorological covariates in the statistical models.

Additionally, the temperature within a thermal image might vary with angle of view, illumination, and distance to targets (Prashar and Jones, 2014). In our experimental design, these parameters were maintained largely constant by a plant monitoring platform. All images were horizontally captured above the seedlings during a short time interval, ensuring a fixed distance to the seedlings. The mixed pixels on the plant edges were removed during the image processing. As all seedlings were morphologically similar, these geometric and lighting factors can also be neglected.

### Provenance performance

The differences in stress sensitivity and recovery of the six provenances were studied after adjusting for PSWD or (more simply) for treatment (water supply). During the stress period, the increased PSWD indicated mild drought stress even in the control group (Figure 1B). Therefore, the provenance performance was studied under two conditions of water stress; extreme (drought treatment) and moderate (control treatment in the stress period).

The Spanish and Bulgarian seedlings were more distressed by over-saturated than under-saturated water conditions (Figure 4). Excessive soil water may decrease the oxygen availability for roots. The lower root respiration reduces the root function and causes leaf dehydration (Vartapetian and Jackson, 1997). Consistent with these findings, the ecophysiological leaf traits of Scots pine (net assimilation, stomatal conductance and transpiration) are lower in short-term water-logged soils than in soils hydrated to field capacity (Repo et al., 2016). The germination rates, shoot growths and root growths of Scots pines of different provenances also respond differently to waterlogging (Mukassabi et al., 2012).

Important triggers of the drought stress response besides PSWD were treatment, provenance and the provenance–PSWD and provenance–treatment interactions. However, because of the limited sample size, significant differences in stress sensitivity between the provenances were rarely identified. Both lines of evidence (PSWD and water supply) imply that pine species' response to strong drought stress (either modeled for 100% PSWD or for the drought treatment during the stress period, when the soil moisture approximated the wilting point) is comparable among provenances. At 100% PSWD, significant differences were observed only between the most extreme provenances (Spain and France, and additionally between France and Poland in the Ig analysis). In contrast, for moderate stress (i.e., over the range of measured PSWD, for the given water supply in the control treatment, or as modeled for 50% PSWD) our study revealed significant differences. Pines in the Spanish provenance tended to be less sensitive to moderate drought stress than Italian pines. Furthermore, the Spanish and Bulgarian pines were significantly less stressed at 50% PSWD than the German and Polish ones, and the Italian provenance demonstrated higher response to mild stress under the control water supply than the French and Bulgarian provenances. Taeger et al. (2013a, 2015) investigated the stem diameter, stem length and respective relative growth rates in pines from these provenances, and reported similar differences among the provenances. The difference in stress levels between the control group (exposed to moderate stress) and the drought group (exposed to extreme drought stress) were smallest and largest in pines from Italy and Spain, respectively (see Supplementary Figure 3).

The Spanish, French, and Italian provenances experience a Mediterranean climate with minimal precipitation in summer; whereas the Bulgarian provenance (despite its similarly dry summer season) is continental (see Supplementary Figure 4 and Table 1). In contrast, the German and Polish provenances are clearly characterized as temperate-continental.

In moderate drought scenarios (50% PSWD or the control group during the stress period in), the pines from provenances with a summer precipitation minimum at their origin (Bulgaria, France, Spain) were more stress-resilient than pines from provenances with rainy summers (Germany, Poland). Thus, the temperate continental provenances exhibited the highest stress under moderate drought. The superior resilience of the Mediterranean type group (the Spanish and Bulgarian provenances) lacks an obvious explanation. Mediterranean Scots pines, which are considered to naturally adapt to drought events (Cregg and Zhang, 2001; Richter et al., 2012; Taeger et al., 2013a), may have adapted their stomatal control under our study conditions. This finding may also imply that southern (Mediterranean) provenances transpired less and used a smaller share of the offered water supply 3050 ml during 42 days of treatment. Tognetti et al. (1997) reported comparable results in drought-stressed *Pinus halepensis*, whose leaf conductance depends on the moisture content of its origin. Drought adaptation in *P. sylvestris* might be governed by lower investment in aboveground biomass and higher biomass allocation to roots (Cregg and Zhang, 2001; Taeger et al., 2015). However, identifying the mechanism of drought adaptation is beyond the scope of this study.

During the recovery period, pines from the Polish, German, and Italian provenances maintained significantly higher stress levels than their Spanish and Bulgarian counterparts. This trend might also reflect a differential stomatal control.

An important feature of our study was the overlap of stress levels in both treatments, despite clear PSWD differences between the treatments. This phenomenon, which appeared in some instances and was especially observed in pines from the Italian provenance, suggests a resource saving strategy as the stress levels were already high under mild drought conditions (control treatment). Such a strategy is supported by a previous mortality experiment, in which trees from the Italian provenance were least threatened by drought-induced mortality (Seidel and Menzel, 2016).

### Influence of seedling dimensions on stress levels

The seedling diameter did not significantly differ among the provenances, but the continental individuals were taller than the Spanish and French ones. Although the seedling dimensions were not included in the final linear mixed-effects models relating PSWD to stress levels, provenance-specific growth traits might contribute to the water responses of individual trees, as mentioned above. The exclusion of tree dimensions from the PSWD models indicates that dimensional traits do not purely drive the observed differences between provenances. This is also indicated by the fact that the water supply models included both provenance and tree dimensions.

In the water supply models, the seedling dimensions (diameter and height) significantly affected the stress response. In particular, the stress levels generally decreased with increasing tree diameter (Figures 5C,F). Trees can store a considerable amount of water in sapwood, whose volume is closely related to stem diameter (Meinzer et al., 2001). Indeed, a simulation study of *Pinus sylvestris* showed a strong relationship between stored water use and tree diameter. Stored water use can contribute up to 40% of the total daily transpiration (Verbeeck et al., 2007). Thus, large diameter provides a water buffer against drought stress. Under mild drought conditions, we detected a positive relationship between stress level and seedling height (Figures 5B,E). A link between reduced growth of aboveground biomass and increased drought adaptation has been suggested (Alía et al., 2001; Valladares et al., 2007). Thus, the increased stress levels in higher seedlings may reflect the higher water consumption of larger sized seedlings or the higher transpiration of larger needle area. In contrast, the seedlings in the drought group exhibited uniform stress levels, most likely because they consumed the irrigation water to an equal extent.

## Conclusion

The study investigated Scots pine specimens from six provenances. In practical forestry, the best provenance for a given water supply (i.e., effective stand precipitation) is an important question. The results suggest a trade-off between stress resistance and height growth; that is, higher stress tolerance is inevitably linked to smaller trees. As the dimensions drive the water consumption of an individual tree, the drought effects are less prominent under nominal soil moisture conditions than under a controlled water supply. In summary, Scots pine seedlings from different provenances respond differently to moderate drought stress, but more uniformly to severe drought stress. Thus, by regarding leaf temperature as a stress indicator, we conclude that drought sensitivity and resilience of Scots pine depends on its native provenance. Individuals from Mediterranean climates, especially from Spain and Bulgaria, are better adapted to moderate drought than pines from temperate continental regions. In practical forestry, provenance-based assisted migration may be a viable adaptation response to climate change.

## Author contributions

HS collected data, contributed to the experimental design, analyzed, and interpreted the data and wrote the paper. CS collected data, contributed to experimental design, revised the paper and contributed to writing the paper. MM contributed to experimental design, contributed to data analyses, revised the paper and contributed to writing the paper. AM contributed to the conception of the work, interpreted the data and wrote the paper.

### Conflict of interest statement

The authors declare that the research was conducted in the absence of any commercial or financial relationships that could be construed as a potential conflict of interest.
