# Supplementary material for: Diverging Drought Resistance of Scots Pine Provenances Revealed by Infrared Thermography
Source: Front Plant Sci. 2016 Aug 31;7:1247. doi: 10.3389/fpls.2016.01247 (PMC5005371; doi:10.3389/fpls.2016.01247)
Supplement: Supplementary file 1 [file DataSheet1.DOCX]

Supplementary Material

Diverging drought resistance of Scots pine provenances revealed by infrared thermography

Hannes Seidel*, Christian Schunk, Michael Matiu, Annette Menzel

*** Correspondence:** hseidel@wzw.tum.de

# Supplementary Methods

## Supplementary Figures


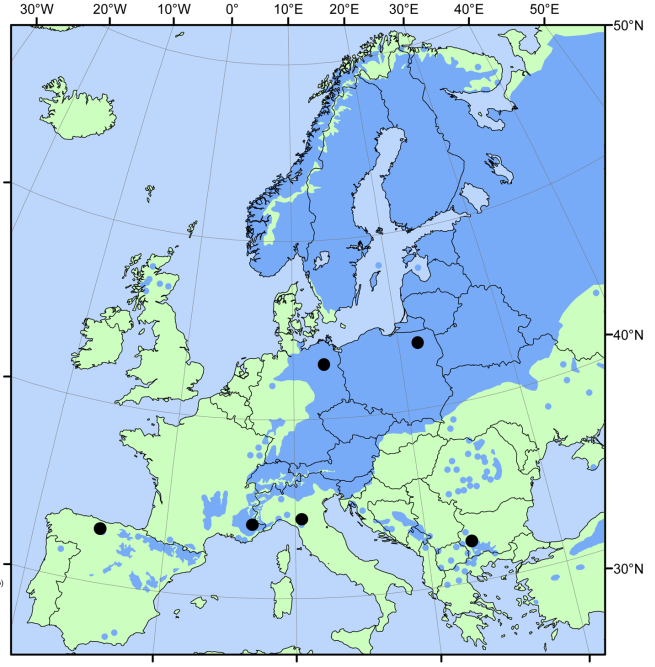


**Supplementary Figure 1. Origin of the six selected provenances across Europe**. Blue area shows the natural distribution of Scots pine (modified after EUFORGEN, 2009)


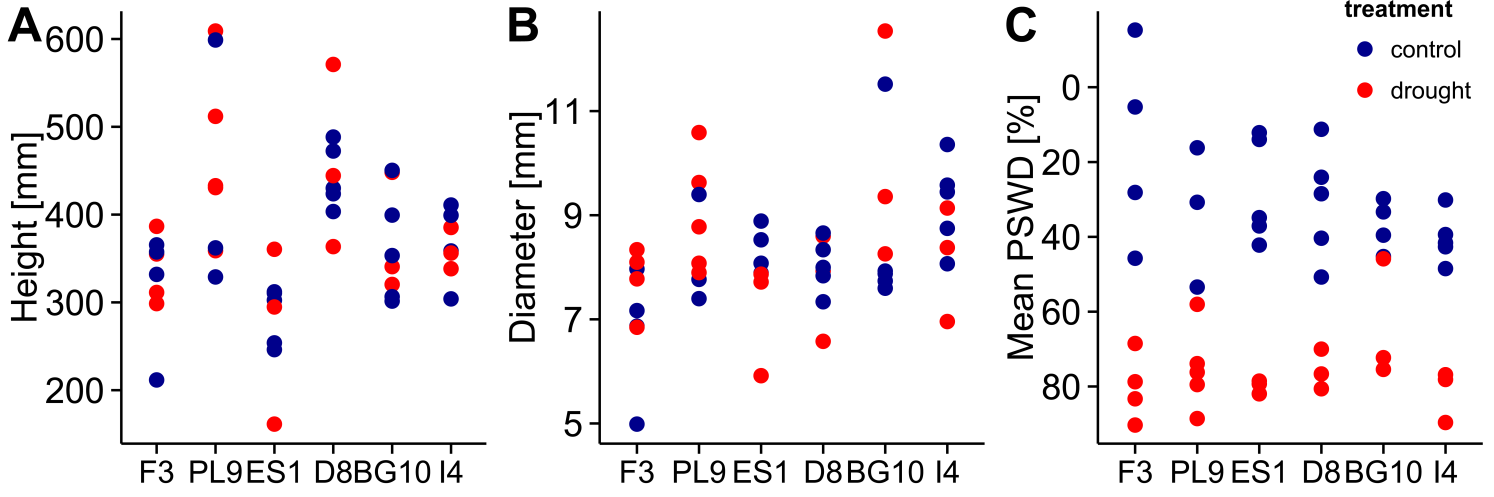


**Supplementary Figure 2. Dimensions and soil water availability of provenances. (**A) Height and (B) diameter of pine individuals of the different provenances before the start of treatment in July 2013. (C) Mean percent soil water deficit (PSWD) per pot during the stress period. Provenance abbreviations are as follows: France (F3), Poland (PL9), Spain (ES1), Germany (D8), Bulgaria (BG10) and Italy (I4).


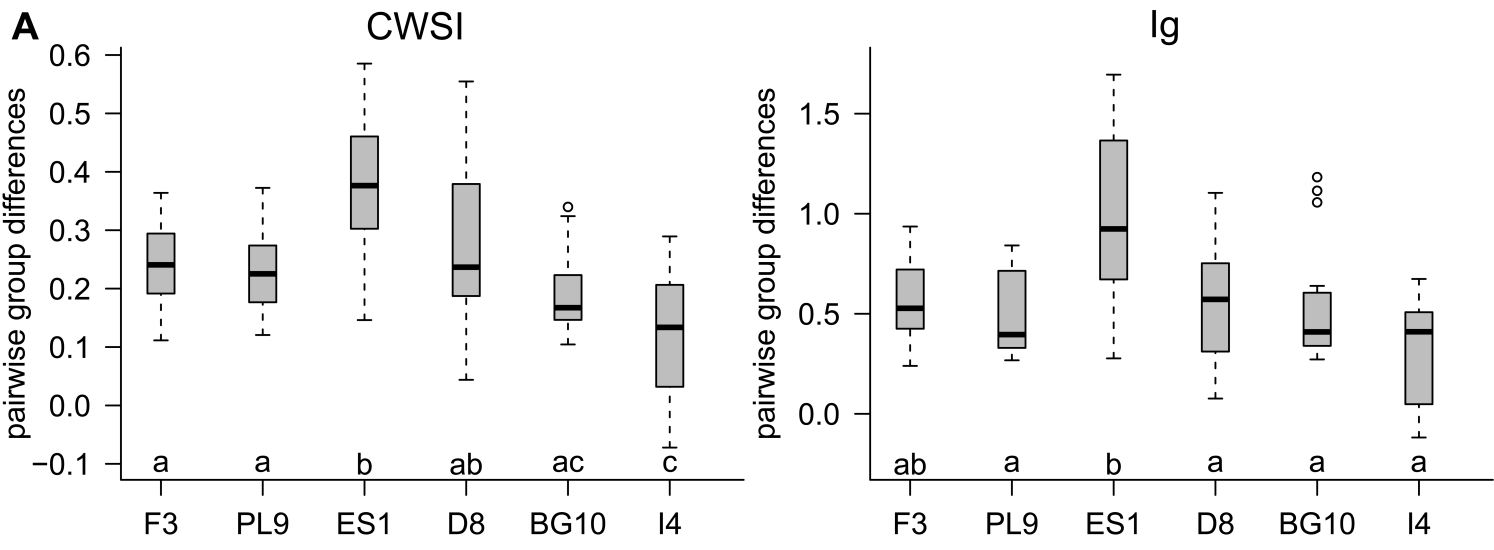


**Supplementary Figure 3. Response of provenances during the stress period (July 17^th^ to August 21^st^).** Responses were calculated within each provenance as the differences of mean values per individual and treatment period between each individual in one experimental group in comparison to all individuals in the other experimental group for the thermal indices (A) crop water stress index (CWSI) and (B) stomatal conductance index (Ig). Responses of provenances that share the same letters below the boxplot are not significantly different at the 5% level. Provenance abbreviations are as follows: France (F3), Poland (PL9), Spain (ES1), Germany (D8), Bulgaria (BG10) and Italy (I4).


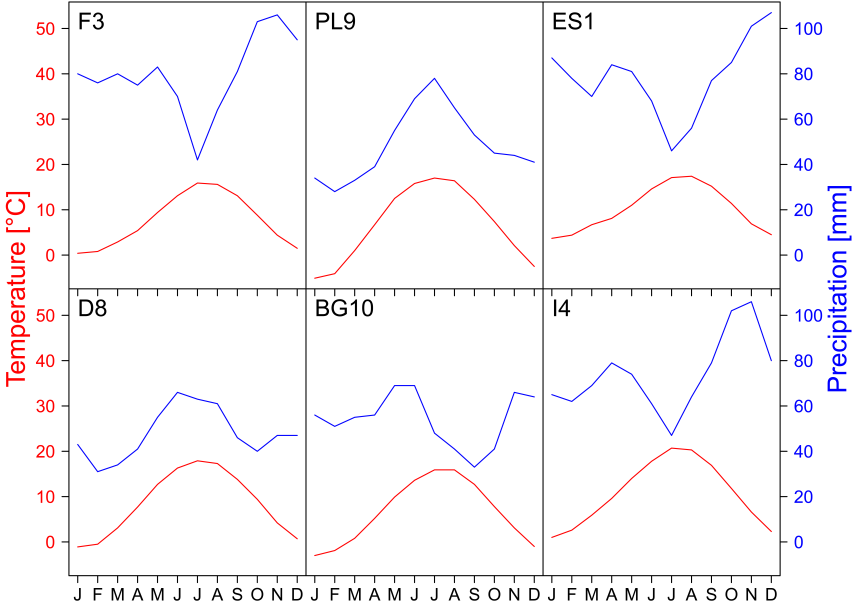


**Supplementary Figure 4. Climatograms at the origin of provenances from France (F3), Poland (PL9), Spain (ES1), Germany (D8), Bulgaria (BG10) and, Italy (I4).**Climate data obtained from the WorldClim data base (Hijmans et al., 2005) for the period 1950 – 2000.

## Supplementary Tables

**Supplementary Table 1. Estimates of thermal indices derived with linear mixed-effects models under different scenarios of percent soil water deficit (PSWD).** Pairwise comparisons of provenances were done using Tukey's range test of contrasts. Values sharing the same letter within a column are significantly different at a level of 0.05. Ig was square-root-transformed. Provenance abbreviations are as follows: France (F3), Poland (PL9), Spain (ES1), Germany (D8), Bulgaria (BG10) and Italy (I4).

|  | CWSI | | | Ig | | |
| --- | --- | --- | --- | --- | --- | --- |
| PSWD | 0% | 50% | 100% | 0% | 50% | 100% |
| F3 | 0.58^ab^ | 0.64^ab^ | 0.81^a^ | 0.74^ab^ | 0.6^ab^ | 0.23^a^ |
| PL9 | 0.68^b^ | 0.72^b^ | 0.97^ab^ | 0.48^b^ | 0.4^b^ | 0.01^b^ |
| ES1 | 0.50^a^ | 0.54^a^ | 1.06^b^ | 1.12^a^ | 0.91^a^ | 0.0^b^ |
| D8 | 0.69^b^ | 0.72^b^ | 0.93^ab^ | 0.43^b^ | 0.38^b^ | 0.03^ab^ |
| BG10 | 0.52^a^ | 0.57^ac^ | 0.94^ab^ | 0.98^a^ | 0.8^ac^ | 0.05^ab^ |
| I4 | 0.61^ab^ | 0.67^bc^ | 0.91^ab^ | 0.67^ab^ | 0.52^bc^ | 0.09^ab^ |

**Supplementary Table 2. Estimated differences of pair-wise comparisons (Tukey's range test of contrasts) during the stress period.** Differences of provenances´ performance under different water supplies (drought treatment and control treatment with moderate stress) as well as for the provenance-specific treatment effect (drought vs. control treatment) estimated with linear mixed-effects models. Ig was square-root-transformed.

|  | Control treatment | | Drought treatment | |
| --- | --- | --- | --- | --- |
| CWSI | Ig | CWSI | Ig |  |
| D8 - BG10 | 0.06 | -0.07 | 0.12 | -0.27 |
| ES1 - BG10 | -0.01 | 0.04 | 0.03 | -0.06 |
| F3 - BG10 | 0.01 | 0.01 | 0.02 | 0.02 |
| I4 - BG10 | 0.16** | -0.27° | 0.02 | 0.00 |
| PL9 - BG10 | 0.06 | -0.09 | 0.13 | -0.26 |
| ES1 - D8 | -0.07 | 0.12 | -0.08 | 0.20 |
| F3 - D8 | -0.05 | 0.08 | -0.10 | 0.29 |
| I4 - D8 | 0.1 | -0.20 | -0.10 | 0.27 |
| PL9 - D8 | 0.00 | -0.02 | 0.02 | 0.01 |
| F3 - ES1 | 0.01 | -0.04 | -0.02 | 0.09 |
| I4 - ES1 | 0.17** | -0.32° | -0.01 | 0.06 |
| PL9 - ES1 | 0.07 | -0.14 | 0.10 | -0.19 |
| I4 - F3 | 0.15* | -0.28 | 0.01 | -0.02 |
| PL9 - F3 | 0.05 | -0.10 | 0.12 | -0.28 |
| PL9 - I4 | -0.10 | 0.18 | 0.11 | -0.26 |
| Drought vs. control treatment | | | | |
| BG10 | 0.23*** | -0.43** |  |  |
| D8 | 0.29*** | -0.63*** |  |  |
| ES1 | 0.27*** | -0.54*** |  |  |
| F3 | 0.24*** | -0.41** |  |  |
| I4 | 0.09 | -0.16 |  |  |
| PL9 | 0.30*** | -0.60*** |  |  |
| Significance codes: ‘***’ 0.001 ‘**’ 0.01 ‘*’ 0.05 ‘º’ 0.1 | | | | |

**Supplementary Table 3. Estimated differences of pair-wise comparisons (Tukey's range test of contrasts) during the recovery period.** Differences between provenances´ performance and differences between water supplies (drought treatment and control treatment with moderate stress). Ig was square-root-transformed.

|  | CWSI | Ig |
| --- | --- | --- |
| D8 - BG10 | 0.20*** | -0.42*** |
| ES1 - BG10 | 0.00 | 0.01 |
| F3 - BG10 | 0.07 | -0.15 |
| I4 - BG10 | 0.11* | -0.22° |
| PL9 - BG10 | 0.14* | -0.27* |
| ES1 - D8 | -0.20*** | 0.43*** |
| F3 - D8 | -0.13* | 0.27* |
| I4 - D8 | -0.09 | 0.20° |
| PL9 - D8 | -0.06 | 0.15 |
| F3 - ES1 | 0.07 | -0.16 |
| I4 - ES1 | 0.11* | -0.23° |
| PL9 - ES1 | 0.14* | -0.28* |
| I4 - F3 | 0.04 | -0.07 |
| PL9 - F3 | 0.07 | -0.13 |
| PL9 - I4 | 0.03 | -0.05 |
| Drought vs. control treatment | | |
|  | -0.11*** | 0.23*** |
| Significance codes: | | |
| ‘***’ 0.001 ‘**’ 0.01 ‘*’ 0.05 ‘º’ 0.1 | | |

**Supplementary Table 4. Z-scores of pairwise comparisons (Dunn´s test) of provenances´ response magnitudes during the stress period (July 17^th^ to August 21^st^) between control and drought treatment estimated with Dunn´s test.** Provenance abbreviations are as follows: France (F3), Poland (PL9), Spain (ES1) , Germany (D8) Bulgaria (BG10) and Italy (I4).

|  | CWSI | Ig |
| --- | --- | --- |
| D8-BG10 | 1.96° | 0.17 |
| ES1-BG10 | 3.97*** | 2.97* |
| ES1-D8 | 2.01° | 2.80* |
| F3-BG10 | 1.57 | 0.67 |
| F3-D8 | -0.43 | 0.50 |
| F3-ES1 | -2.47* | -2.35° |
| I4-BG10 | -1.12 | -1.42 |
| I4-D8 | -3.08* | -1.58 |
| I4-ES1 | -5.09*** | -4.38*** |
| I4-F3 | -2.71* | -2.11° |
| PL9-BG10 | 1.29 | -0.40 |
| PL9-D8 | -0.67 | -0.57 |
| PL9-ES1 | -2.68* | -3.37** |
| PL9-F3 | -0.26 | -1.07 |
| PL9-I4 | 2.41* | 1.02 |
| Significance codes: | | |
| ‘***’ 0.001 ‘**’ 0.01 ‘*’ 0.05 ‘º’ 0.1 | | |

## Supplementary References

EUFORGEN (2009). Distribution map of Scots pine (*Pinus sylvestris*). *www.euforgen.org.*

Hijmans, R. J., Cameron, S. E., Parra, J. L., Jones, P. G., and Jarvis, A. (2005). Very high resolution interpolated climate surfaces for global land areas. *Int. J. Climatol.* 25, 1965–1978. doi:10.1002/joc.1276.
